# Contrasting Role of Temperature in Structuring Regional Patterns of Invasive and Native Pestilential Stink Bugs

**DOI:** 10.1371/journal.pone.0150649

**Published:** 2016-02-29

**Authors:** P. Dilip Venugopal, Galen P. Dively, Ames Herbert, Sean Malone, Joanne Whalen, William O. Lamp

**Affiliations:** 1 Department of Entomology, University of Maryland, College Park, Maryland, United States of America; 2 Tidewater Agricultural Research and Education Center, Virginia Polytechnic Institute and State University, Suffolk, Virginia, United States of America; 3 Department of Entomology and Wildlife Ecology, University of Delaware, Newark, Delaware, United States of America; Universidad Nacional Autonoma de Mexico, MEXICO

## Abstract

**Objectives:**

Assessment and identification of spatial structures in the distribution and abundance of invasive species is important for unraveling the underlying ecological processes. The invasive agricultural insect pest *Halyomorpha halys* that causes severe economic losses in the United States is currently expanding both within United States and across Europe. We examined the drivers of *H*. *halys* invasion by characterizing the distribution and abundance patterns of *H*. *halys* and native stink bugs (*Chinavia hilaris* and *Euschistus servus*) across eight different spatial scales. We then quantified the interactive and individual influences of temperature, and measures of resource availability and distance from source populations, and their relevant spatial scales. We used Moran’s Eigenvector Maps based on Gabriel graph framework to quantify spatial relationships among the soybean fields in mid-Atlantic Unites States surveyed for stink bugs.

**Findings:**

Results from the multi-spatial scale, multivariate analyses showed that temperature and its interaction with resource availability and distance from source populations structures the patterns in *H*. *halys* at very broad spatial scale. *H*. *halys* abundance decreased with increasing average June temperature and distance from source population. *H*. *halys* were not recorded at fields with average June temperature higher than 23.5°C. In parts with suitable climate, high *H*. *halys* abundance was positively associated with percentage developed open area and percentage deciduous forests at 250m scale. Broad scale patterns in native stink bugs were positively associated with increasing forest cover and, in contrast to the invasive *H*. *halys*, increasing mean July temperature. Our results identify the contrasting role of temperature in structuring regional patterns in *H*. *halys* and native stink bugs, while demonstrating its interaction with resource availability and distance from source populations for structuring *H*. *halys* patterns.

**Conclusion:**

These results help predicting the pest potential of *H*. *halys* and vulnerability of agricultural systems at various regions, given the climatic conditions, and its interaction with resource availability and distance from source populations. Monitoring and control efforts within parts of the United States and Europe with more suitable climate could focus in areas of peri-urban developments with deciduous forests and other host plants, along with efforts to reduce propagule pressure.

## Introduction

Biological introductions cause major environmental changes globally and amongst the introduced species, herbivorous insects especially cause severe ecological and economic damages [1–3]. Assessment and identification of the spatial structures in populations of introduced biota is an important step towards unraveling the ecological processes that structure them [4,5]. By understanding spatial variation in the distribution and abundance of the invasive species, and relating them to abiotic and biotic factors, macroecological patterns and processes driving their invasion could be derived [6–8]. Such knowledge, especially those spanning multiple spatial and temporal scales for both the introduced and native biota, helps elucidate factors driving invasion process and formulation of effective, long term management strategies [9–11].

The brown marmorated stink bug, *Halyomorpha halys* (Stål 1855) (Hemiptera: Pentatomidae) originally from east Asia, was detected in 1990s and confirmed in 2001 near Allentown, Pennsylvania [12] in north eastern United States. Since then it has spread to 41 different States, and also expanded into Canada [13]. It is a severe agricultural pest in the mid-Atlantic region and nuisance pest elsewhere entering homes for overwintering [14]. In Europe, *H*. *halys* populations are established in Switzerland and its range has expanded into Germany, Liechtenstein, France, Italy, Hungary and Greece [15,16]. With increasing interceptions in Australia [17] and New Zealand [18], *H*. *halys* could become a global invasive pest [15,16].

*Halyomorpha halys* is a polyphagous stink bug with over 120 host plants including tree fruits, vegetables, field crops, ornamental plants, and forest trees in its native and invaded ranges [14,15,19]. Since 2010, serious crop losses are reported in mid-Atlantic United States for apples (*Malus domestica* Baumg.; $37million), peaches [*Prunus persica* (L.) Batsch], sweet corn and field corn (*Zea mays* L.), capsicum (*Capsicum annuum* L.), tomatoes (*Solanum lycopersicum* L.), and soybeans [*Glycine max* (L.)]. Other common native stink bugs in the mid-Atlantic agricultural systems are the green stink bug, *Chinavia hilaris* (Say 1832), and the brown stink bug, *Euschistus servus* (Say 1832), both economically important pests of crops in the south and southeastern United States [20,21]. However, *H*. *halys* is now the most abundant stink bug pest in the agricultural fields of the mid-Atlantic region [22,23].

Invasion success is a function of the interactions among climatic tolerances, host availability, and propagule pressure in the context of anthropogenic influences [7,11,24–26]. Particularly, insects are highly sensitive to temperature which affects all life history parameters including development, abundance and distribution (e.g. Law of Tolerance; [27,28]). Studies on *H*. *halys* patterns at large spatial scales and on factors affecting their distribution and abundance are few. Zhu et al. [29] and Haye et al. [15] highlighted the importance of temperature while estimating the global spatial distribution of *H*. *halys* by matching climatic conditions between native and invaded ranges.

In southeastern and parts of mid-Atlantic United States, Bakken et al. [30] identified the pattern of increasing abundance of *H*. *halys* along with increasing altitude, a surrogate for decreasing temperature. Wallner et al. [31] identified the role of urban developments and resource availability (host plants in natural habitats and agricultural regions) during different stages of the invasion process, and in influencing *H*. *halys* abundance. To understand its invasion success, we need assessments of multi-scale spatial structures in *H*. *halys* distribution and abundance, and knowledge on the interactive role of climate, resources availability and propagule pressure. Such spatially explicit analyses may help identify areas with high risk of *H*. *halys* invasion and inform spatially targeting resources and control strategies [7,9,11].

In this study, we follow the thematic recommendations by McIntire & Fajardo [32] and employ ‘space as a surrogate’ explanatory variable to discern the ecological processes driving the spatial structures in invasive and native stink bug pests. We formulated various hypotheses that combined correlational and mechanistic approach, incorporating biological processes to the modeling procedure. The hypotheses were 1) spatial structures are absent and *H*. *halys* populations are randomly distributed, representing null hypothesis; 2) spatial structures observed at very broad scales primarily in relation to climatic conditions since temperature profoundly influences *H*. *halys* development, growth, survival and abundance [33–36]; 3) natural habitats and developed areas provide both food and overwintering resources [14,19,37] and may influence *H*. *halys* abundance at multiple spatial scales (broad to medium; dependent on the scale they themselves vary over the region); 4) dispersal from adjacent habitats [23,38] and intraspecific and interspecific interactions such as semio-chemically mediated aggregations [39,40] or egg parasitism by parasitoids [41] influence abundance at fine to very fine spatial scales and 5) a combination of exogenous factors (climate and resource availability) with endogenous factors (dispersal and species interactions) could affect abundance at multiple spatial scales (broad and fine spatial scales).

Utilizing data from a regional network of sampled soybean fields, we tested these hypotheses for *H*. *halys* and such similar hypotheses for *C*. *hilaris* and *E*. *servus*. We addressed the specific questions—a) at which spatial scales are the abundance of the invasive and native stink bug species in the mid-Atlantic region structured?; b) what are the primary environmental (bioclimatic, surrogate measures of resource availability, and distance from original source population) factors associated with invasive and native stink bug abundance, and at which spatial scales do they operate?; c) do the spatial structures persist after the environmental influences have been accounted for, and at which spatial scales?; and d) what are the individual and interactive influences of the significant environmental (bioclimatic, measures of resource availability, and distance from source population) and spatial variables on the observed patterns of *H*. *halys* and native stink bugs?

## Methods

### Ethics Statement

No endangered or protected species were involved in the study. We obtained permission from numerous individual private farmers for access and data collection in their soybean fields.

### Study area and stink bug sampling

**Fig 1:**
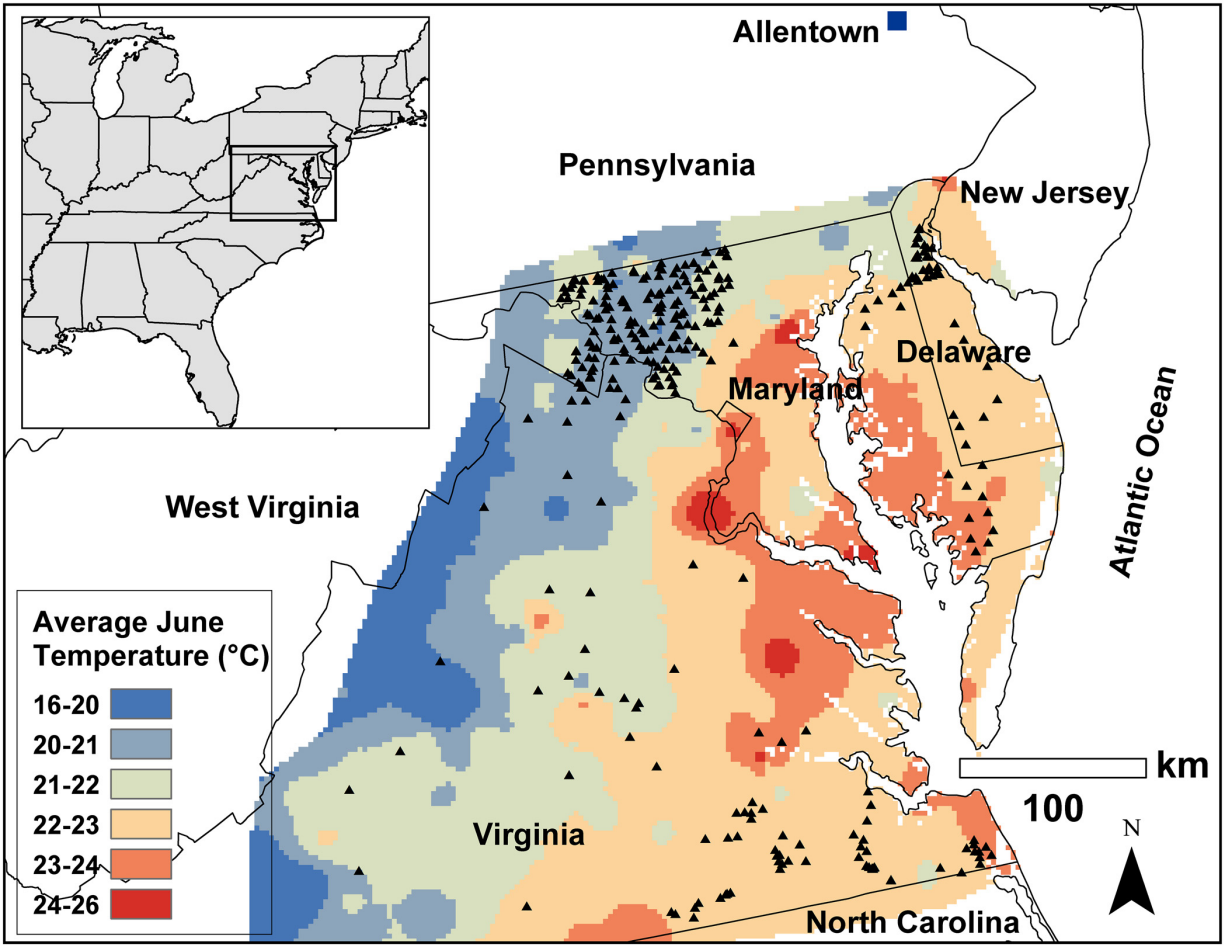
Location of soybean fields sampled (triangles) for stink bugs across different States and source of original *Halyomorpha halys* population (Allentown) in mid-Atlantic USA. Map also depicts the gradient of average monthly temperature (°C) in June 2013 based on interpolations of temperature data from 430 weather stations in the study area freely available at Climate Data Online (http://www.ncdc.noaa.gov/cdo-web/search?datasetid=GHCNDMS). The inset map shows the location of the study area (bounded by rectangle) in eastern USA. We generated the maps with spatial data on administrative state boundaries available freely through United States Census Bureau (https://www.census.gov/geo/maps-data/data/tiger-line.html).

The study was conducted across a large portion of mid-Atlantic United States (36.5°–39.7° N and 75.3°–78.8° W) in Delaware, Maryland, Virginia and West Virginia (Fig 1) encompassing gradients of topographical and bioclimatic conditions, and heterogeneous landscapes. *H*. *halys* has been a serious agricultural pest in the study area for the past 5 years and soybean was chosen as focal crop because it is a preferred host of *H*. *halys* both in its native and introduced ranges [19]. Stink bug sampling was conducted between Aug 25–Sep 16 of 2012 and 2013, coinciding with peak *H*. *halys* abundance in soybean during the seed development stages in the mid-Atlantic region [38]. To avoid biases in abundances due to soybean maturity differences among fields we only sampled in full season soybean fields (late May–early June plantings), devoid of any insecticidal applications. Stink bug abundance was determined by sweep net (38 cm diameter) sampling in the soybean canopy within 5m from field edge, where stink bug densities typically are the highest [23,38]. We took a set of 25 sweeps at each of three sides of each field. To ensure uniformity, field crew members maintained similar sweep net procedures (sweeping height, speed and sweep net arc length; [42]). The species (*H*. *halys*, *C*. *hilaris* and *E*. *servus*) and number of stink bugs observed in the sweep net samples were either recorded in the field or collected for lab identification and enumeration. We also noted the spatial coordinates for each of the 329 soybean fields sampled (111 fields in 201, and 218 in 2013).

### Explanatory variables

We used topography, temperature, proximal distance from source population, and quantitative measures of landscape composition (henceforth land use) as variables explaining stink bug distribution and abundances across the study area. We calculated 72 explanatory variables—four topographical, four temperature, 63 land use variables, and a variable with distance to *H*. *halys* source location (S1 and S2 Tables). Topography was characterized by altitude, and slope and aspect were derived with digital elevation models from National Elevation Dataset [43]. We further transformed the aspect values using trigonometric functions (sine and cosine) to identify ‘eastern’ and ‘northern’ exposures respectively. We derived average monthly temperatures at sampled fields for May, June, July and August through inverse distance weighted interpolations of daily minimum and maximum temperatures during 2012 and 2013 at 430 weather stations within the study area [44]. We calculated the Euclidean distance of each sampled field to the proximal location of *H*. *halys* introduction and source population within the United States (Allentown, Pennsylvania; [12]).

Data on land use surrounding the sampled fields were generated by overlaying and clipping buffers from CropScape data layer [45–47]. Land use variables were measured across a range of spatial scales for accurate identification of species-land use associations [48]. We quantified land use at seven different spatial scales (100m, 250m, 500m, 1000m, 2500m, 5000m and 10000m radii centered in sampled fields) on the basis of *H*. *halys* dispersal capability and its land use association [23,31,49,50]. For each spatial scale, we derived the proportion of land use in nine different categories of crop, forest and developed areas (see S2 Table for details). All spatial data manipulations and compilations for the landscape, topography and climatic variables were performed in ArcGIS 10.2 [51].

### Statistical Analyses

The analytical procedures broadly follows that of the worked example in Dray et al. [5]. For each sampled field, we used the overall abundance for each species summed across the three sweep net for analysis. The sampled field by species abundance table was transformed to emphasize abundant (*H*. *halys*) and rare species (*C*. *hilaris* & *E*. *servus*) using the Hellinger and chi-square transformations, respectively [5,52]. We analysed main patterns in *H*. *halys* and native stink bugs through individual principal component analysis (PCA) on each of these tables. We used redundancy analysis (RDA) to identify the influence of topography, temperature, distance from source (for *H*. *halys*) and multi-scale land use on observed patterns in stink bugs. Prior to RDA we applied forward selection procedures with two-stop criteria to both the transformed data tables [53] and retained only the respectively important explanatory variables. We performed partial residual analysis (PRA; partial principal component analysis with environmental factors as covariates) to understand patterns in the unexplained variance, the residual data from RDA of both tables.

The spatial component was quantified using Moran’s Eigenvector Maps (MEMs) based on Gabriel graph [54]. We used the MEM framework [5,55] to estimate and test the multi-scale components of spatial patterns in each of the transformed initial data tables focusing on *H*. *halys* or the native stink bugs, their approximation with environmental variables, and the residual variance tables. We computed scalograms for each table by projecting the site scores of the first two axes of the respective ordination analyses (PCA, RDA and PRA) onto the spatial context defined by the MEMs (328 each for *H*. *halys* and native species tables). Thereby, we partitioned the respective variances into spatial scales spanning from broadest to the finest. A smoothing procedure [56] was applied to the scalograms, dividing them into 8 spatial components each with 41 successive MEMs constituting a gradient of very broad (entire study area) to very fine spatial scales (single to few sampled soybean fields). The individual scalogram R^2^ values (measure of the amount of variation explained by a given spatial scale) are expected to be uniformly distributed when spatial structure is absent [5,57]. Through a permutation procedure, as demonstrated by [5], we tested if the maximum observed R^2^ (R^2^ max; spatial scale of smoothed MEM at which the stink bug pattern is mainly structured) is significantly higher than the values determined in the absence of a spatial pattern.

We assessed the relative importance and interactive influences of the significant environmental and spatial variables on the observed stink bug patterns using multivariate variation partitioning [58,59]. Prior to this analysis, we applied separate forward selection procedures with two-stop criteria [53] to the MEMs spatial variables for both data tables, and selected only the significant MEMs. We further divided the selected MEMs into broad-medium scales (MEMs associated with positive Moran’s *I* statistic), and fine scales (negative Moran’s *I* statistic). The statistical significance of the various fractions of the partitioned variation was determined through permutation tests with 999 random permutations and we provide the adjusted *R*^*2*^ values (*R*^*2*^_a_).

PCA, RDA and PRA, and the permutation tests were performed using package ‘ade4’ [60]. MEMs were generated with package ‘spacemakeR’ [61] and package ‘spdep’ [62] was used for the Gabriel graph. Scalogram computations and, smoothing and permutation procedures were performed by modifying source codes from Dray et al. [5]. Forward selection of variables were performed with package ‘packfor’ [63]; variation partitioning and permutation tests were performed using package ‘vegan’ [64] and its results were plotted using package ‘Vennerable’ [65] all in R program [66].

## Results

In the sweep net samples, 9942 individuals of the three stink bug species were recorded of which *H*. *halys* and *C*. *hilaris* respectively constituted 85% and 11%. The maximum abundance of *H*. *halys* at a field was 283 per 75 sweeps, and it was not present in 99 of 329 sampled soybean fields.

### *Halyomorpha halys* spatial patterns

**Fig 2:**
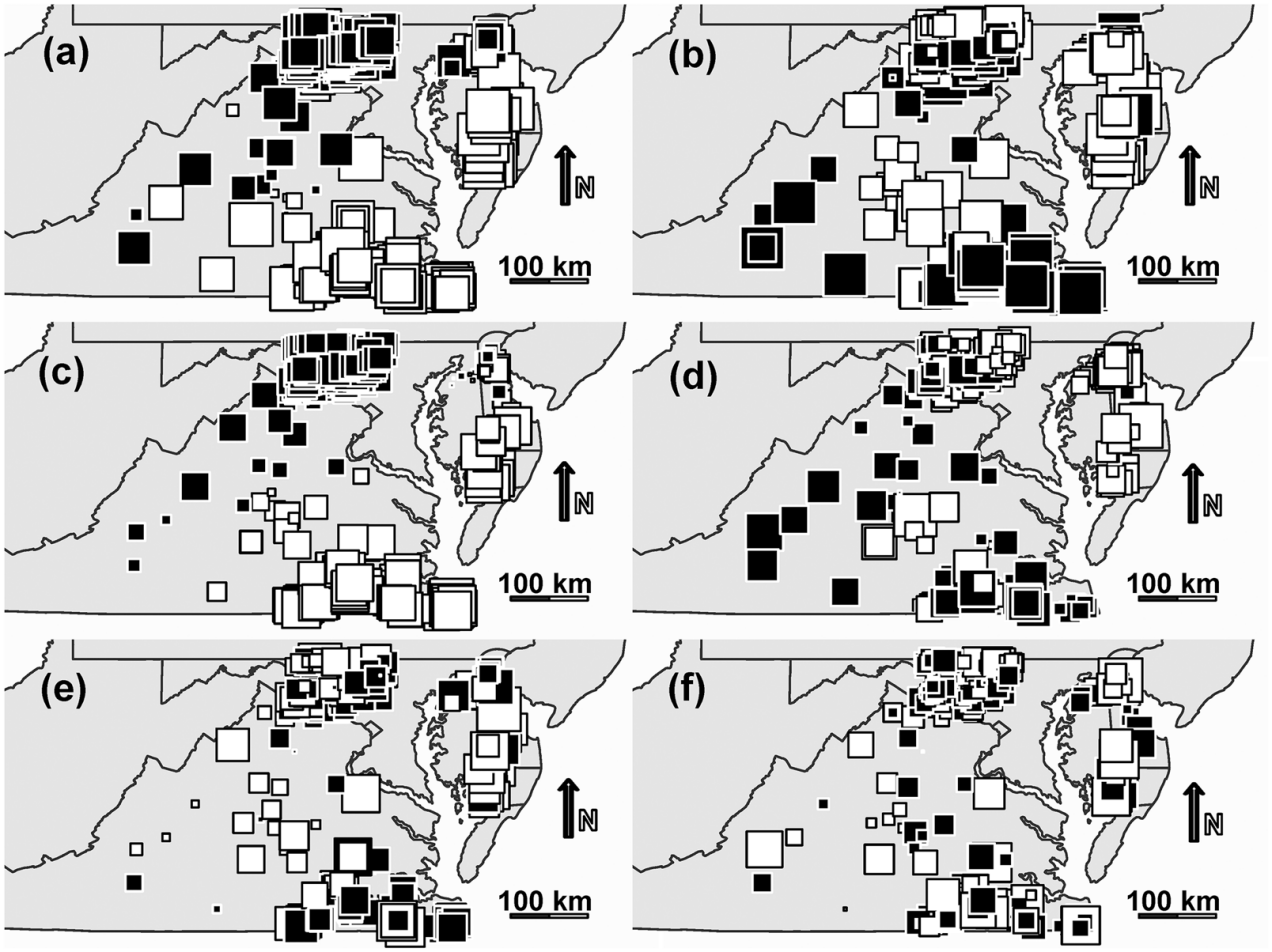
Maps of plot scores from the first two axes of PCA (a, b), approximation with explanatory variables through RDA (c, d), and the residual data analysis PRA (e, f). Analyses were performed on Hellinger transformed soybean stink bug data from 329 fields in mid-Atlantic USA, emphasizing patterns in the invasive *Halyomorpha halys*. The *black squares* and the *white squares* indicate positive and negative plot scores respectively. The size of the squares is proportional to its score, the farther from zero being larger. We generated the maps with spatial data on administrative state boundaries available freely through United States Census Bureau (https://www.census.gov/geo/maps-data/data/tiger-line.html).

**Fig 3:**
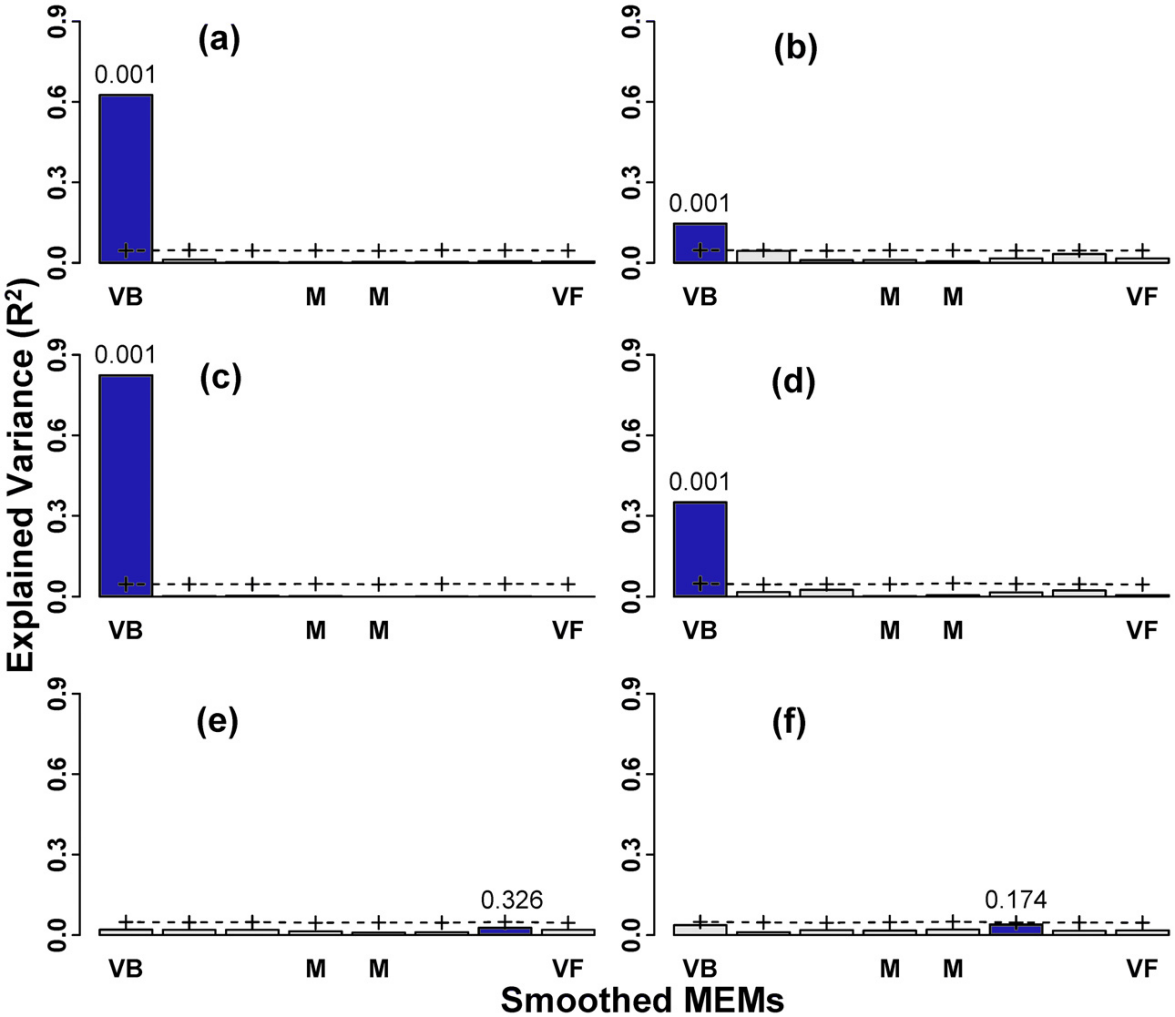
Smoothed scalograms for the Moran’s Eigenvector Maps (MEMs; 328 MEMs assembled in 8 groups) indicating the portion of variance (R^2^adj) explained by each spatial scale for stink bug data emphasizing patterns in *Halyomorpha halys* from soybean fields of mid-Atlantic USA. Scalograms for first two axes of PCA (a, b), approximation with explanatory variables through RDA (c, d), and the residual data analysis PRA (e, f) are provided. The letters VB—Very Broad, M—Medium, and VF—Very fine, denote the spatial scales. For each scalogram, the scale corresponding to the highest R^2^ (in dark blue) was tested using 999 permutations of the observed values, and its p-values are provided. The dotted line with (+) symbols represent the 95% confidence limit.

The overall variation in Hellinger transformed data, i.e. patterns of *H*. *halys*, was represented by PCA axes 1 and 2 which respectively accounted for 56% and 25% of the total variance, and fields with high *H*. *halys* abundance received positive principal components scores along PCA axis 1 (Fig 2a). *H*. *halys* abundance was structured at very broad spatial scales, as revealed by scalograms of the first two PCA axes (Fig 3a and 3b) with significant non-random spatial pattern (axis 1—*R*^2^ Max = 0.63, *p* = 0.001; axis 2—*R*^2^ Max = 0.15, *p* = 0.001). This reflected in the PCA plot scores with a clear spatial trend along east-west orientation for axis 1 (negative scores in eastern portion; Fig 2a).

**Fig 4:**
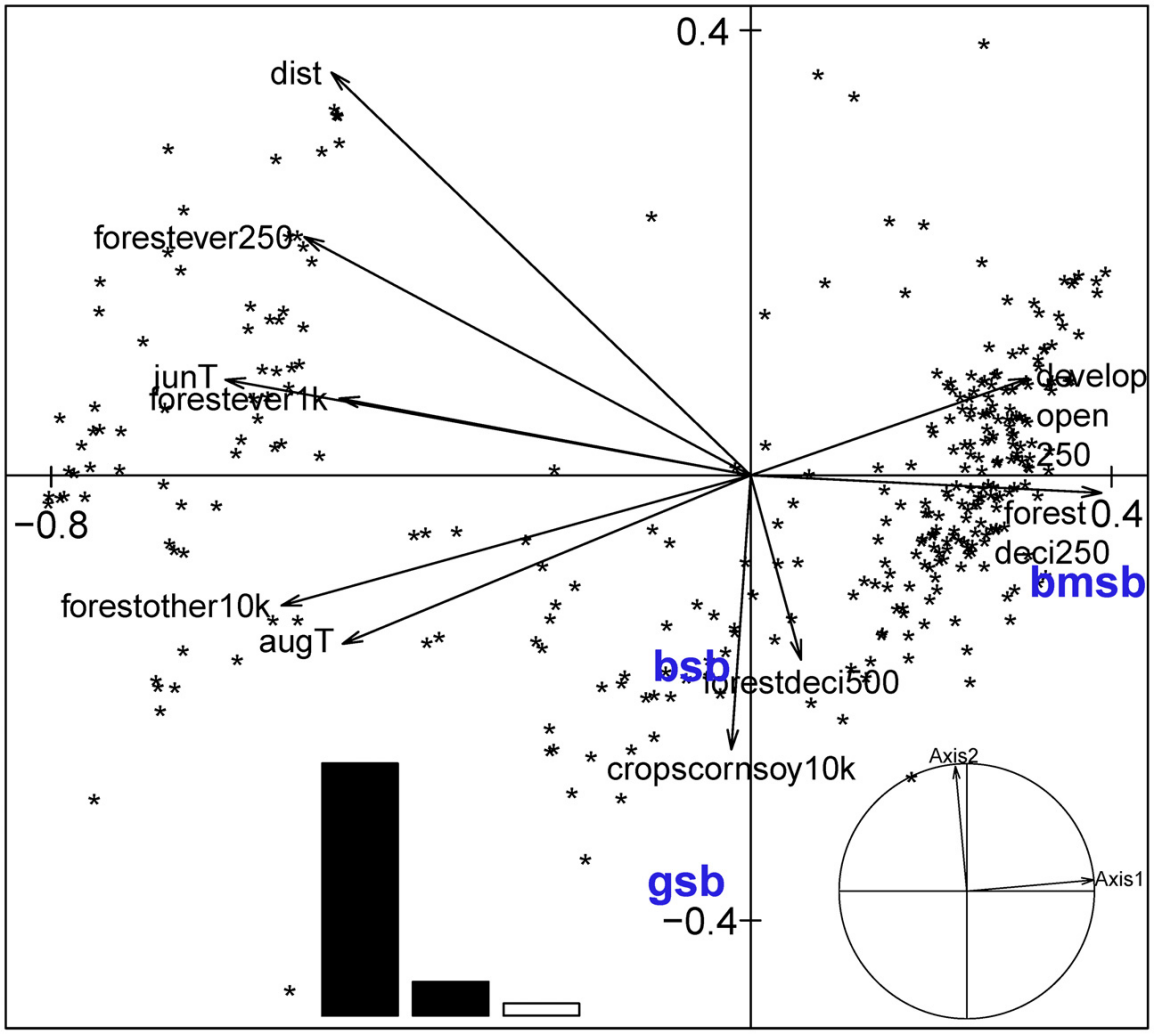
Patterns in *Halyomorpha halys* in relation to environmental variables depicted as biplot of RDA with Hellinger transformed stink bug data from soybean fields of mid-Atlantic USA. The stink bug species include *H*. *halys* (bmsb), *Chinavia hilaris* (gsb) and *Euschistus servus* (bsb). The explanatory variables are average monthly temperatures in June (junT) and August (augT); land use variables–% evergreen forest cover at 250m and 500m (forestever250 and forestever500 respectively), % other natural habitat types at 10km (forestother10km), % deciduous forest cover at 250m and 500m (forestdeci250 and forestdeci500 respectively), and % developed area (open) at 250m (devopen250); and distance from source population (dist). The inset figures depict the inertia of RDA axes (bar graphs), and their eigenvalues.

Forward selection of Hellinger transformed table identified 10 important explanatory variables (Table 1), which explained a significant proportion of variation in *H*. *halys* patterns (RDA; *R*^*2*^_a_ = 0.44, *p* = 0.001). The approximated RDA table showed first two axes respectively accounting for 85% and 12% of the total explained variance. The first RDA axis (Fig 4), pertaining to patterns of *H*. *halys*, was primarily driven by the gradient in average monthly temperature during June (*r* = −0.86) and % other forests at 10 km (*r* = −0.77), and *H*. *halys* abundance (reflected by positive scores along PCA axis 1) decreased with increasing average June temperature (S1 Fig). Fields with high *H*. *halys* abundance were positively associated with % developed open area at 250m (typically large-lot single family houses and vegetation in developed settings) and % deciduous forests at 250m (Fig 4). The temperature, distance from source and land use variables explained the variation in *H*. *halys* predominantly at very broad spatial scales (Fig 3c and 3d; scalogram of RDA axis 1—*R*^2^ Max = 0.82, *p* = 0.001; axis 2—*R*^2^ Max = 0.35, *p* = 0.001), manifested through the non-random spatial patterns in the axes scores (Fig 2c and 2d).

**Table 1:** Important temperature, land use and distance from source variables selected by the forward selection procedure for explaining *Halyomorpha halys* distribution and abundance.

variable code	variable details	order	R^2^	R^2^Cum	AdjR^2^ Cum	F	pval
junT	average temperature for June (°C)	63	0.29	0.29	0.28	131.0	0.001
augT	average temperature for August (°C)	2	0.06	0.34	0.34	28.7	0.001
dist	Euclidean distance of sampled fields from Allentown, Pennsylvania, potential source of *Halyomorpha halys* (km)	46	0.03	0.37	0.37	14.7	0.001
developopen250	% developed open areas at 250m scale	43	0.02	0.39	0.39	11.0	0.001
forestever1k	% evergreen forests at 1 km scale	57	0.02	0.41	0.40	9.3	0.001
forestdeci250	% deciduous at 250m scale	52	0.01	0.42	0.41	8.2	0.001
forestother10k	% all other natural habitat types at 10 km scale	67	0.01	0.44	0.42	6.2	0.001
forestdeci500	% deciduous at 500m scale	53	0.01	0.44	0.43	5.5	0.002
forestever250	% evergreen forests at 250m scale	59	0.01	0.45	0.44	3.7	0.011
cropscornsoy10k	% corn and soybean fields at 10 km scale	5	0.01	0.46	0.44	3.7	0.012

**Fig 5:**
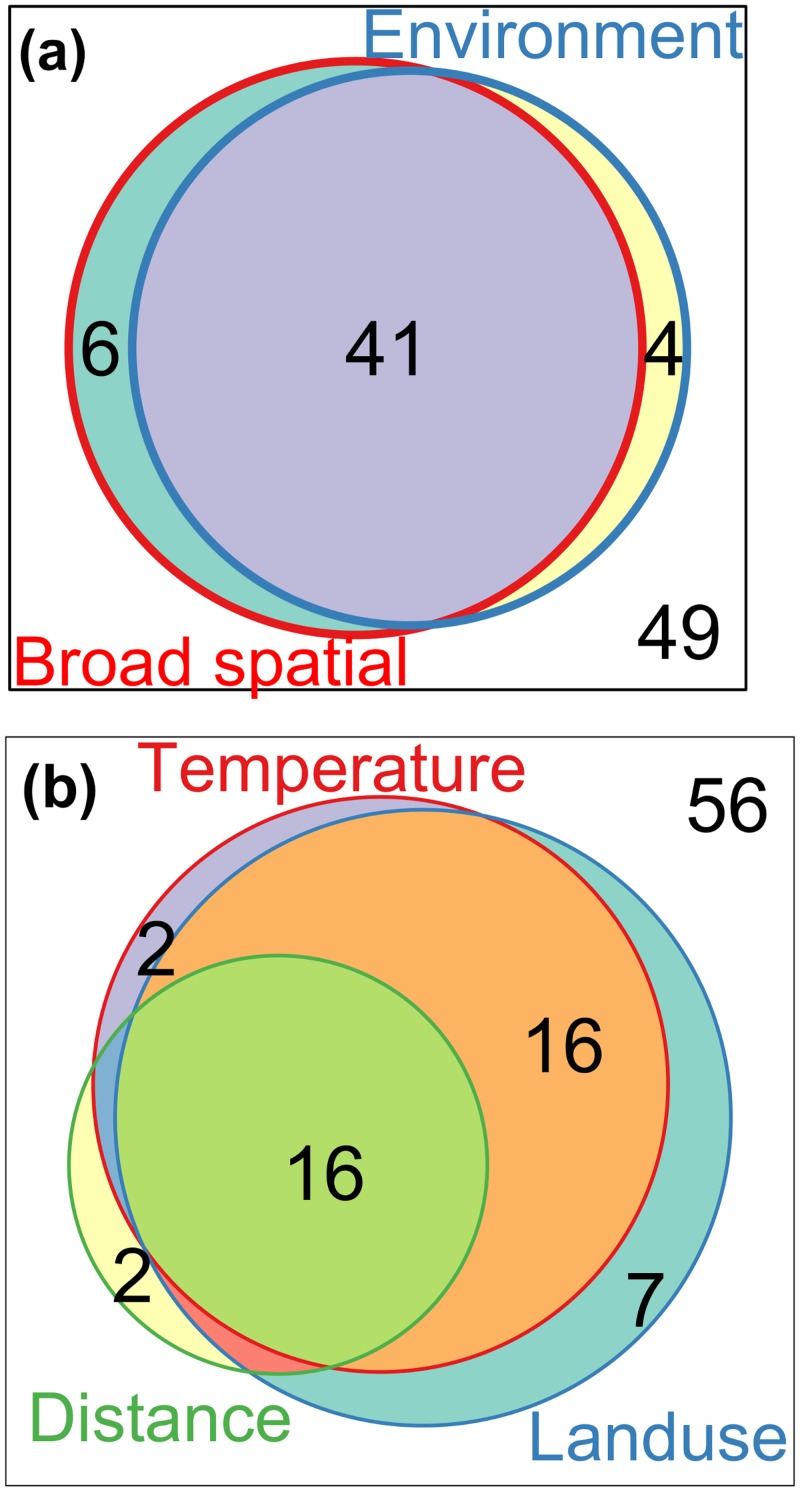
Partitioning the variation in patterns of *Halyomorpha halys* using broad-medium scale Moran’s Eigen Vector spatial component and environmental attributes (a) and, three subsets of environmental data—temperature, land use and distance from source population (b). For each image, the enclosing box represents the total variation in composition, circles within the box approximately indicate the fraction of variation explained by each variable, and value outside the circles represents unexplained variance. All the testable model fractions (i.e. the unique contributions) were significant with *P* < 0.01 after 999 permutations.

Scalograms for axis 1 and 2 of PRA of the residual table did not identify any significant spatial components (Fig 2e and 2f). This implies a lack of spatial trend in the residual data after accounting for the influence of the explanatory variables on *H*. *halys* patterns (Fig 3e and 3f). The forward selection procedure applied to the MEM spatial variables identified 10 MEMs accounting for 47% of the total variation in Hellinger transformed data table, all corresponding to broad-medium scales (positive Moran’s statistic). Variation partitioning with spatial and environmental variables (Fig 5a) identified a prominent fraction constituting of temperature, resource availability, and propagule pressure variables structured at broad-medium scale (*R*^*2*^_a_ = 0.41, *p* = 0.005), significant pure broad-medium scale spatial (*R*^*2*^_a_ = 0.06, *p* = 0.005) and pure environmental fractions (*R*^*2*^_a_ = 0.04, *p* = 0.005). Variation partitioning with just the environmental variables revealed the important interactive influences of climate, resource availability and propagule pressure (*R*^*2*^_a_ = 0.16, *p* = 0.005), and climate and resource availability interaction (*R*^*2*^_a_ = 0.16, *p* = 0.005; Fig 5b) in explaining *H*. *halys* patterns. Additionally, minor, yet significant individual fractions of resource availability (*R*^*2*^_a_ = 0.07, *p* = 0.005), propagule pressure (*R*^*2*^_a_ = 0.02, *p* = 0.005) and temperature (*R*^*2*^_a_ = 0.02, *p* = 0.005) was also observed.

### Spatial patterns of native stink bugs

For the native stink bugs *C*. *hilaris* and *E*. *servus*, a PCA of the chi-square transformed data table constrained most of the overall variation within the first two prominent axes (axis 1–56% and axes 2–38%). Distribution and abundance of native stink bug species revealed patterns at very broad spatial scales (scalograms in S2a and S2b Fig) with significant non-random spatial pattern of the axes scores (S3a and S3b Fig).

**Fig 6:**
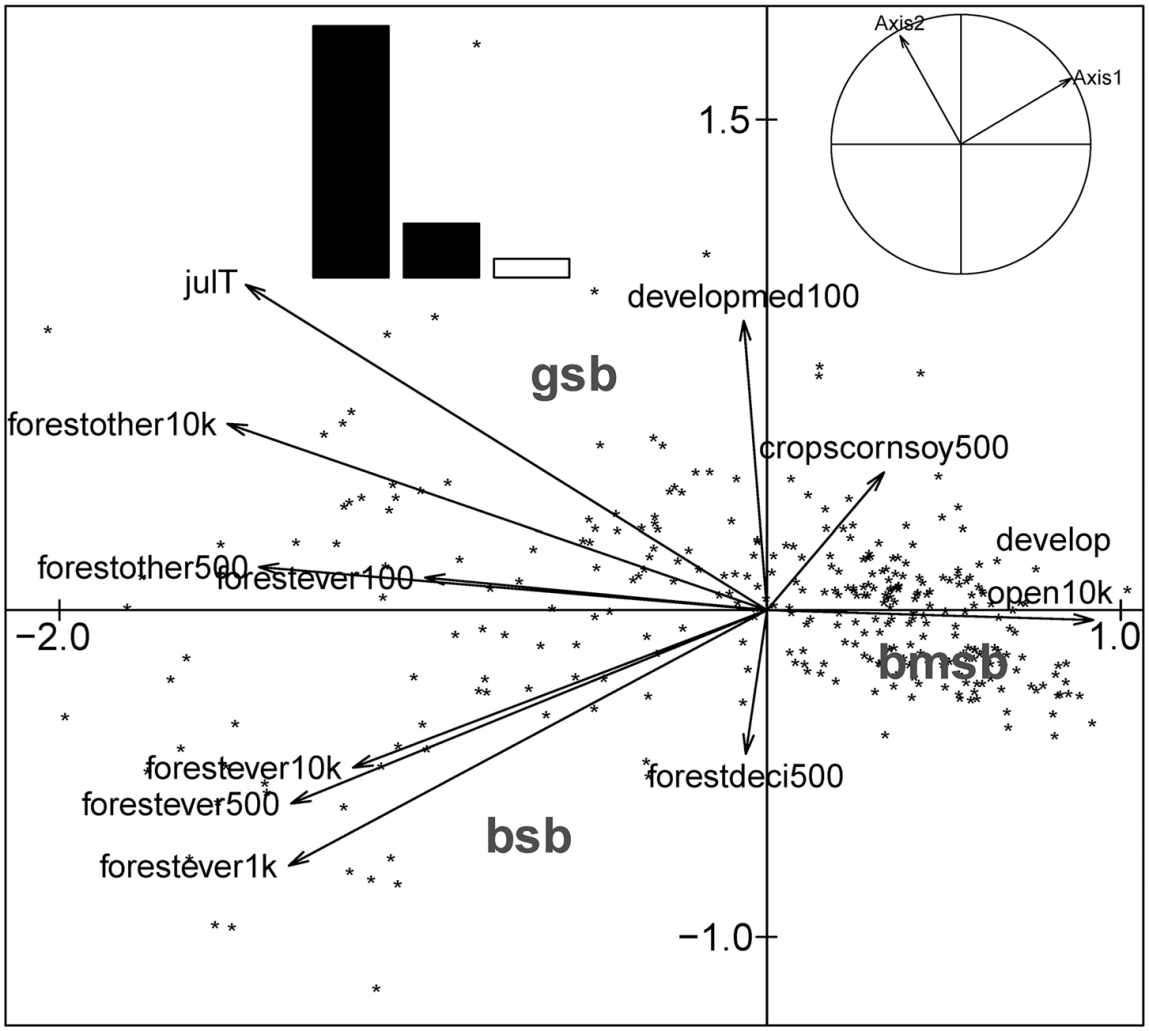
Patterns in *Chinavia hilaris* (gsb) and *Euschistus servus* (bsb) in relation to environmental variables depicted as biplot of RDA for data from soybean fields of mid-Atlantic USA. The explanatory variables include average monthly temperatures in July (julT); and land use variables–% evergreen forest cover at 100m, 500m, 1 km and 5km (forestever100, forestever500, forestever1k and forestever5k respectively), % natural habitat types at 100m (forestother100), % deciduous forest cover at 500m (forestdeci500, % developed area (open) at 10km (devopen5k), % developed area (medium) at 100m and % corn and soybean fields at 500m (cropscornsoy500). The inset plots depict the eigenvalues (bar graph) and the inertia of RDA axes.

Forward selection of chi-square transformed table identified 11 important explanatory variables (Table 2), and through RDA a significant proportion of the variation in stink bug community was explained by these temperature and land use variables (*R*^*2*^_a_ = 0.26, *p* = 0.001). The two prominent RDA axes respectively accounted for 78% and 17% of the total explained variance and RDA axis 1 score was correlated with monthly temperatures during July (*r* = −0.70 with Axis 1), % other forest types at 10 km (*r* = −0.73) and 500m scales (*r* = −0.73) and % evergreen forest cover at 1km (*r* = −0.64) and 500m (*r* = −0.64) scales. Both *E*. *servus* and *C*. *hilaris* were positively associated with perc. forest cover and monthly average temperatures during July (Fig 6) which explained the variation in native stink bug abundances at very broad spatial scales (S2c and S2d Fig), with a non-random spatial pattern in the axes scores (S3c and s3d Fig). For the residual data, scalograms for axis 1 and 2 of PRA did not reveal any significant spatial structures (S2e, S2f, S3e and S3f Figs).

**Table 2:** Important temperature and land use variables selected by the forward selection procedure for explaining *Chinavia hilaris* and *Euschistus servus* distribution and abundance.

variables	variable details	order	R^2^	R^2^Cum	AdjR^2^ Cum	F	pval
forestother10k	% all other natural habitat types at 10 km scale	66	0.121	0.121	0.119	45.105	0.001
forestever1k	% evergreen forests at 1 km scale	56	0.070	0.192	0.187	28.369	0.001
julT	average temperature for July (°C)	61	0.016	0.207	0.200	6.502	0.003
forestever10k	% evergreen forests at 10 km scale	55	0.016	0.223	0.214	6.570	0.002
forestdeci500	% deciduous forest at 500m scale	52	0.012	0.235	0.223	5.015	0.016
developopen10k	% developed open areas at 10km scale	40	0.011	0.246	0.232	4.731	0.010
forestever100	% evergreen forests at 1 km scale	54	0.010	0.256	0.240	4.142	0.026
forestother500	% all other natural habitat types at 500m scale	70	0.009	0.265	0.246	3.974	0.024
forestever500	% evergreen forests at 1 km scale	59	0.007	0.272	0.252	3.272	0.031
developmed100	% medium developed areas at 10km scale	32	0.008	0.280	0.257	3.340	0.037
cropscornsoy500	% corn and soybean fields at 500m scale	9	0.006	0.286	0.262	2.849	0.048

**Fig 7:**
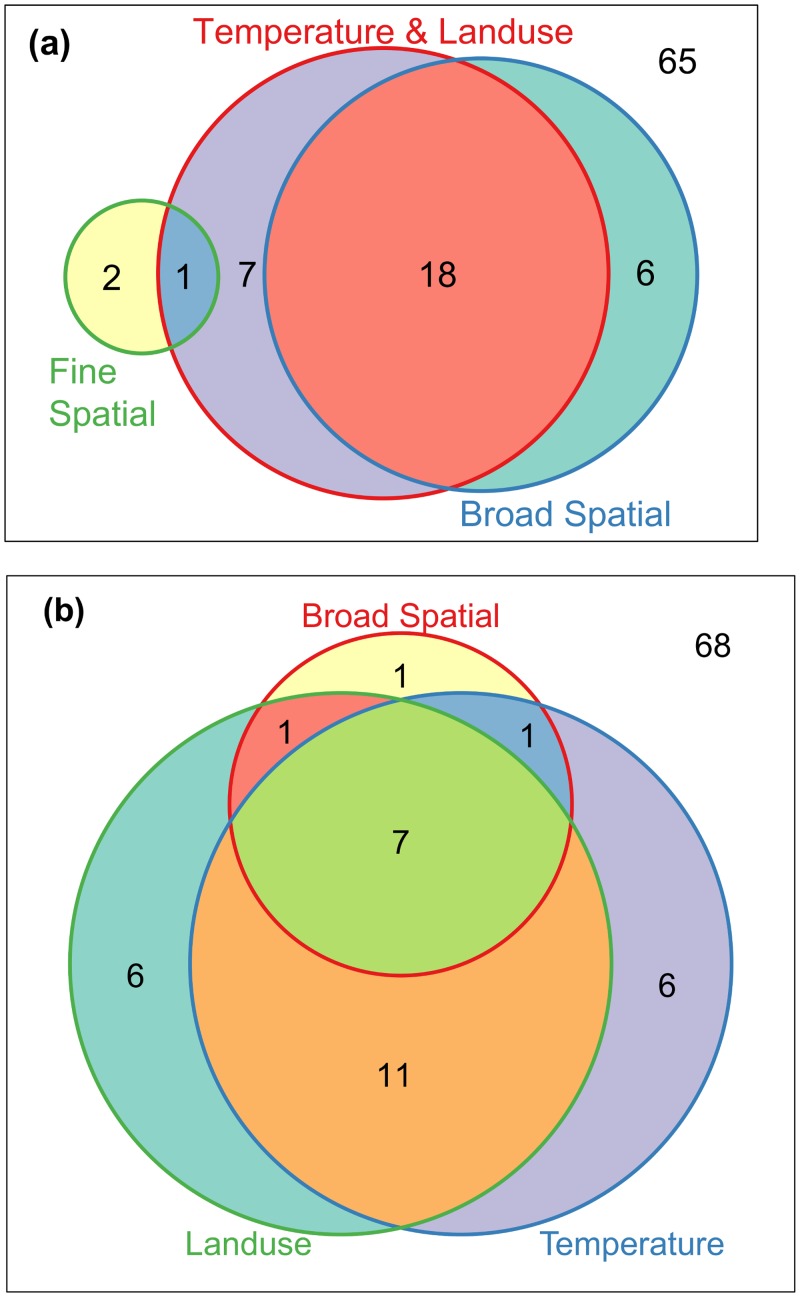
Partitioning the proportions of variation in patterns of *Chinavia hilaris* and *Euschistus servus* explained by (a) environmental variables (land use and temperature), broad and fine scale Moran’s Eigenvector Maps (MEMs) spatial components, and (b) environment (land use and temperature) and broad scale MEM spatial component. For each image, the enclosing box indicates the total variation in composition, circles within the box approximately indicate the fraction of variation explained by each of the variables, and values outside the circles represent unexplained variance. All the testable model fractions (i.e. the unique contributions) were significant with *P* <0.01 after 999 permutations.

The forward selection procedure applied to the MEM spatial variables for native stink bug data identified nine MEMs accounting for 27% of the total variation in chi-square transformed data table, corresponding to both broad and fine scales (seven and two MEMs with positive and negative Moran’s statistic respectively). Variation partitioning revealed the prominent influence of environmental factors structured at broad scales on the native stink bug patterns observed (*R*^*2*^_a_ = 0.18, *p* < 0.01; Fig 7a), along with the individual influences of pure environment (*R*^*2*^_a_ = 0.7, *p* < 0.01) and pure broad scale spatial factors (*R*^*2*^_a_ = 0.6, *p* < 0.01). The overall influence of fine scale spatial attributes on native stink bug patterns were low, but a significant pure fine scale spatial fraction was observed (*R*^*2*^_a_ = 0.2, *p* < 0.01). Variation partitioning with broad spatial, temperature and resource availability variables (Fig 7b) revealed prominent interactive (*R*^*2*^_a_ = 0.11, *p* < 0.01), and their individual influences (*R*^*2*^_a_ = 0.06, *p* < 0.01 for both).

## Discussion

Our results elucidate the predominant role of temperature in structuring regional stink bug patterns at very broad spatial scales. Our results also identify the interactive influences of temperature, resource availability and distance from source population on producing the broad scale spatial patterns. Presence of spatial structures at very broad scales highlight the predominant role of environment (mainly temperature) in structuring regional stink bug patterns. On the other hand, spatial structures arising due to due to biotic processes are expected to occur at medium to fine spatial scales [5,54,67–69]. The lack of spatial patterns at fine scales suggests that the influence of biotic processes such as semio-chemical aggregation [40] or dispersal [49,50,70] on the abundances of *H*. *halys* and the native stink bugs may be localized (fine scales). We did not document or quantify biotic processes but our results suggest that for both the invasive *H*. *halys* and native stink bugs, macroclimate (temperature) structures local biotic interactions [71–73]. Land use features such as developed area are important estimators of *H*. *halys* abundance at finer spatial scales (than temperature), but are not the primary drivers of *H*. *halys* patterns at the regional scale. Temperature is the most important abiotic factor that affects herbivorous insects such as stink bugs by directly influencing their development, survival, distributional range and abundance [74].

### Contrasting role of temperature on *Halyomorpha halys* and native stink bug patterns

*H*. *halys* abundance was negatively associated with increasing temperature during June, the early part of reproductive season [22]. Egg mass and early stadia of *H*. *halys* are particularly vulnerable at around the lower and upper temperature thresholds (15–17°C and 33–35°C respectively) and lab studies indicate 25°C as the optimal temperature for survival and development of different stadia [34,36]. However, our results revealed a strong negative relationship between *H*. *halys* abundance and average temperatures in June above 23.5°C, and *H*. *halys* were not recorded at fields with average June temperature higher than 23.5°C (see S1 Fig). This is probably due to cumulative mortality of eggs and earlier stadia at prolonged exposure closer to upper temperature threshold. Also, while previous reports document the role of spring temperature on *H*. *halys* pre-oviposition behaviour [36] and overall population survival [35], spring temperature (average May temperature) did not influence *H*. *halys* abundance in our study.

Contrastingly, the abundances of the native stink bugs *C*. *hilaris* and *E*. *servus* increased with average July temperature. With a distributional range across the United States [20], these stink bugs may have higher temperature tolerances than the invasive *H*. *halys*. For example, *C*. *hilaris* survivorship at 27°C for second stadia and adults were 88% and 80% respectively [75], and optimal temperature for this species was estimated as 28.4°C [76] (but see [77]). Similar to the contrasting influence of temperature in our study, Menke et al. [78] found that, at the landscape scale, the invasive Argentine ant *Linepithema humile* (Mayr, 1868) occurrence and the native ant diversity were respectively positively and negatively correlated to minimum winter temperatures.

The positive association of *H*. *halys* abundance with urbanized areas is consistent with earlier reports from mid-Atlantic region [31]. Vegetation and ornamental trees near dwellings, and deciduous forests provide primary food resources for adults emerging from overwintering and their offspring [22]. In particular, the introduced [*Paulownia tomentosa* (Thunb.) Steud. and *Ailanthus altisimma* (Mill.) Swingle], native and ornamental trees (*Acer* L., *Prunus* L., *Pyrus* L., and *Malus* Mill.) are preferred host plants for the first generation [22,30]. Also, structures (houses and other buildings) in urban settings, and deciduous forest trees are ideal overwintering sites for *H*. *halys* both in its native Asia and the invaded regions [19,37]. In contrast, the native North American stink bugs *C*. *hilaris* and *E*. *servus* were primarily associated with forested areas which contain many woody host plants such as *Prunus serotina* Ehrhart, *Sambucus canadensis* L., and *Quercus* L. [21,79], and also they overwinter in the forest floor [80].

### Implications for understanding *Halyomorpha halys* invasion and agricultural management

Our results will help to predict the establishment, spread, and pest potential of *H*. *halys* in invaded regions, and for management of stink bugs in agricultural systems. The interactive effect of temperature, resource availability, and distance from source populations we report here is similar to that of 29 most invasive quarantine European arthropods [26], Guatemalan potato tuber moth, *Tecia solanivora* (Povolný 1973), in Ecuador [81] and the invasive Argentine ant [82]. The interactive influences are important for establishment and growth of populations already spread to other parts of United States beyond our study area (e.g. west coast of United States) and in Europe. The interactive influences, primarily structured by temperature, also explain the patterns of higher adult *H*. *halys* abundance in pheromone traps in inland eastern United States, than the north Atlantic coastal plains or Pacific Northwest [39]. For the mid-Atlantic region containing high field crop acreage in the eastern portion, if temperatures during June are similar to our study period (2012–2013), high abundances of *H*. *halys* might not occur. Monitoring, control and management efforts in such conditions could focus on the north western portion (Piedmont province; [83] of our study area (proximity of source population) particularly in crop fields in peri-urban areas.

In the eastern north central and central climatic regions of United States [84] which contain very high soybean acreage, temperatures during March–June are below or close to the lower thresholds and not optimal for growth and development of *H*. *halys* eggs and early nymphal stages. However, urbanized areas in these regions could support high abundance of *H*. *halys* by acting as ‘heat islands’ that provide refuge [85] facilitating high pest abundance. Monitoring and management efforts for these regions could focus in agricultural fields in urban and peri-urban areas. Similarly, for the south east climatic region where temperatures are warmer than the mid-Atlantic, *H*. *halys* might occur in higher abundance in the western mountains and Piedmont region than in the eastern coastal plains and recent reports identify such patterns [30,39]. Monitoring and control strategies could target peri-urban areas of the Piedmont province and western mountainous regions in the southeast. Finally, the native stink bugs *C*. *hilaris* and *E*. *servus*, economic pests of cotton, wheat, soybean and corn in the southern and southeastern portion of United States, were primarily associated with forest cover and control efforts could focus in fields in rural areas with higher forest cover than in urban and peri-urban areas.

Overall, our study identified the predominant role of temperature and its interaction with resource availability and propagule pressure in structuring regional patters of stink bug abundances. The lack of any clear spatial pattern in the residual data, after accounting for the effect of explanatory variables, indicates that our results are robust. The residual variance in abundance may be the product of species responses to unmeasured environmental drivers, other biotic processes or random stochasticity [5]. For example, other localized variables including cropping patterns, rotations, planting dates, soybean row spacing, soybean variety maturation date, land preparation patterns, and cultivation systems ([86] also influence stink bug abundance. The significant pure spatial components observed in the variation partitioning (broad spatial scales for *H*. *halys* and finer scales for the native species) indicate that the explanatory power of our analyses can be improved further. Temperature during the previous winter and early spring (March and April [35]) could influence *H*. *halys* abundance and relative humidity is important for stink bug egg emergence and dispersal [87]. Additionally, measures of transportation networks (e.g. proximity to ports, road network) might better represent propagule pressure than the distance from source we used in our analyses. Future investigations on *H*. *halys* could include these variables to improve our understanding of the factors and mechanisms facilitating or impeding the distribution and abundance of this invasive agricultural pest.

## Supporting Information

S1 Fig Temperature and *Halyomorpha halys* relationship. Relationship between average June temperature and (a) principal component scores along PCA axis 1 and, (b) the raw abundances of *Halyomorpha halys* across a network of soybean fields in the mid-Atlantic United States.
(TIF)

S2 Fig Scalograms for the native stink bug species. Smoothed scalograms for the Moran’s Eigenvector Maps (MEMs; 328 MEMs assembled in 8 groups) indicating the portion of variance (R^2^) explained by each spatial scale for *Chinavia hilaris* and *Euschistus servus* from soybean fields of mid-Atlantic USA. Scalograms for first two axes of PCA (a, b), approximation with explanatory variables through RDA (c, d), and the residual data analysis PRA (e, f) are provided. The letters VB—Very Broad, M—Medium, and VF—Very fine, denote the spatial scales. For each scalogram, the scale corresponding to the highest R^2^ (in dark grey) is tested using 999 permutations of the observed values, and its p-values are provided. The dotted line with (+) symbols represent the 95% confidence limit.
(TIFF)

S3 Fig Multivariate analyses plot scores for native stink bugs. Maps of plot scores from the first two axes of PCA (a, b), approximation with explanatory variables through RDA (c, d), and the residual data analysis PRA (e, f). Analyses were performed on chi-square transformed soybean stink bug data emphasizing patterns in *Chinavia hilaris* and *Euschistus servus* from 329 soybean fields in mid-Atlantic USA. The black and the white squares indicate positive and negative plot scores respectively. The size of the squares is proportional to its score, the farther from zero being larger.
(TIF)

S1 Table Details on climatic and topographic variables. Description and summary (mean values, and standard deviation in parentheses) of the topographic and temperature variables at sampled soybean fields used for explaining stink bug distribution and abundance in mid-Atlantic US.
(DOCX)

S2 Table Details on land use variables. Description and summary statistics of (mean values and standard deviation in parentheses) the land use variables at different spatial scales used for explaining stink bug distribution and abundance in mid-Atlantic US.
(DOCX)
