# Supplementary figures and images for: Contrasting Role of Temperature in Structuring Regional Patterns of Invasive and Native Pestilential Stink Bugs
Source: PLoS One. 2016 Feb 29;11(2):e0150649. doi: 10.1371/journal.pone.0150649 (PMC4771716; doi:10.1371/journal.pone.0150649)

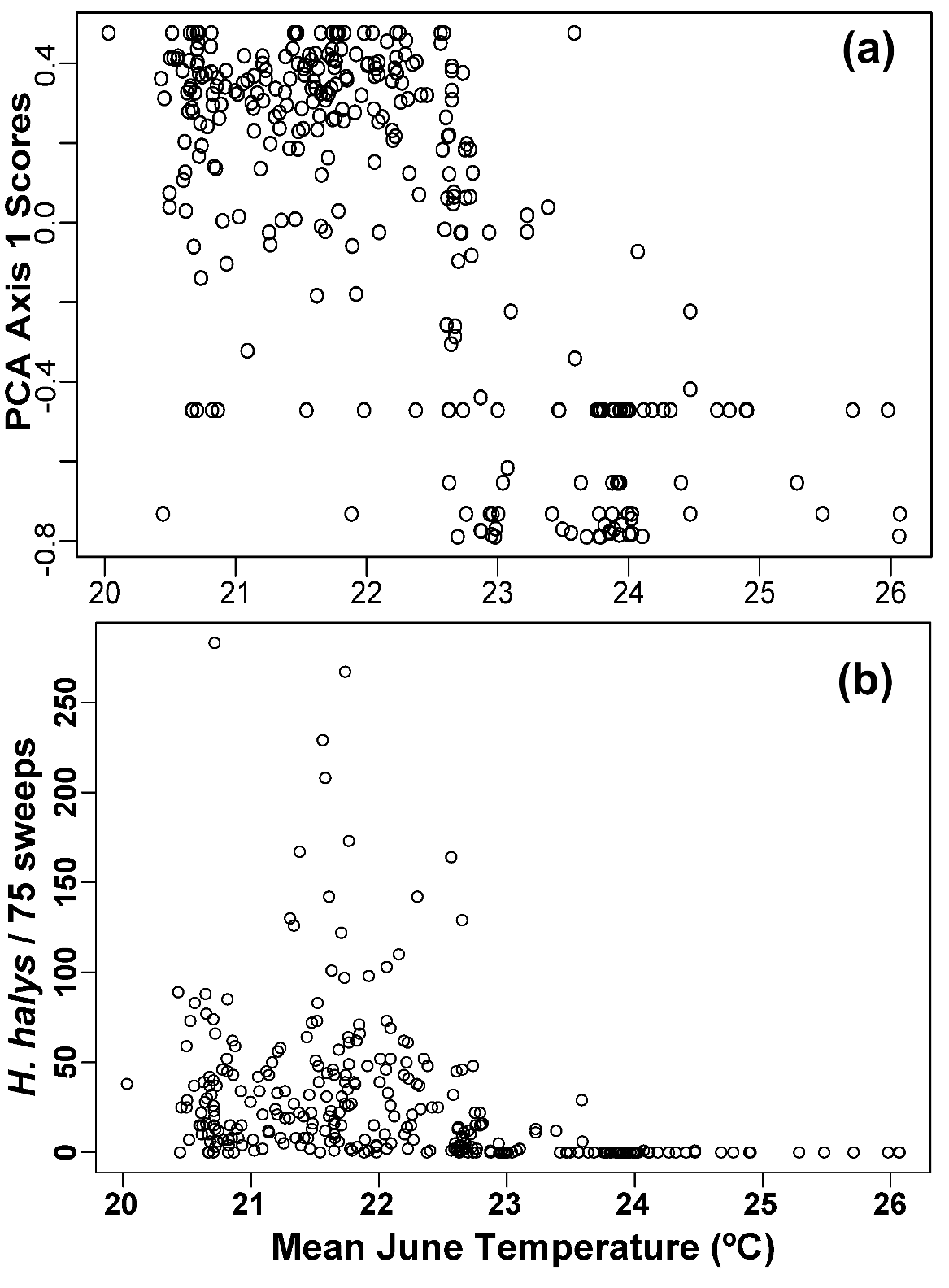

Supplement: S1 Fig — Relationship between average June temperature and (a) principal component scores along PCA axis 1 and, (b) the raw abundances of Halyomorpha halys across a network of soybean fields in the mid-Atlantic United States. (TIF) [file pone.0150649.s001.tif]

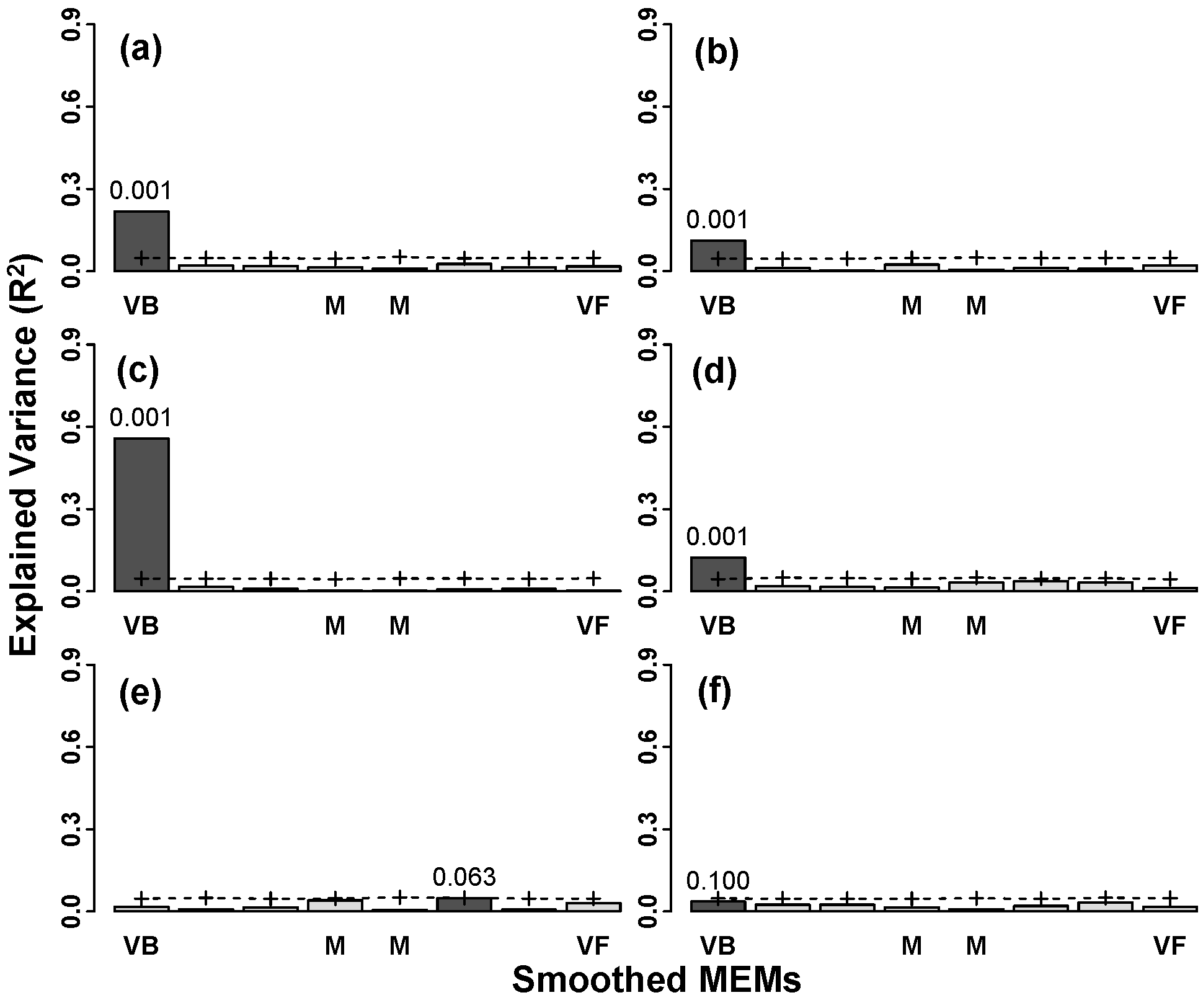

Supplement: S2 Fig — Smoothed scalograms for the Moran’s Eigenvector Maps (MEMs; 328 MEMs assembled in 8 groups) indicating the portion of variance (R2) explained by each spatial scale for Chinavia hilaris and Euschistus servus from soybean fields of mid-Atlantic USA. Scalograms for first two axes of PCA (a, b), approximation with explanatory variables through RDA (c, d), and the residual data analysis PRA (e, f) are provided. The letters VB—Very Broad, M—Medium, and VF—Very fine, denote the spatial scales. For each scalogram, the scale corresponding to the highest R2 (in dark grey) is tested using 999 permutations of the observed values, and its p-values are provided. The dotted line with (+) symbols represent the 95% confidence limit. (TIFF) [file pone.0150649.s002.tiff]

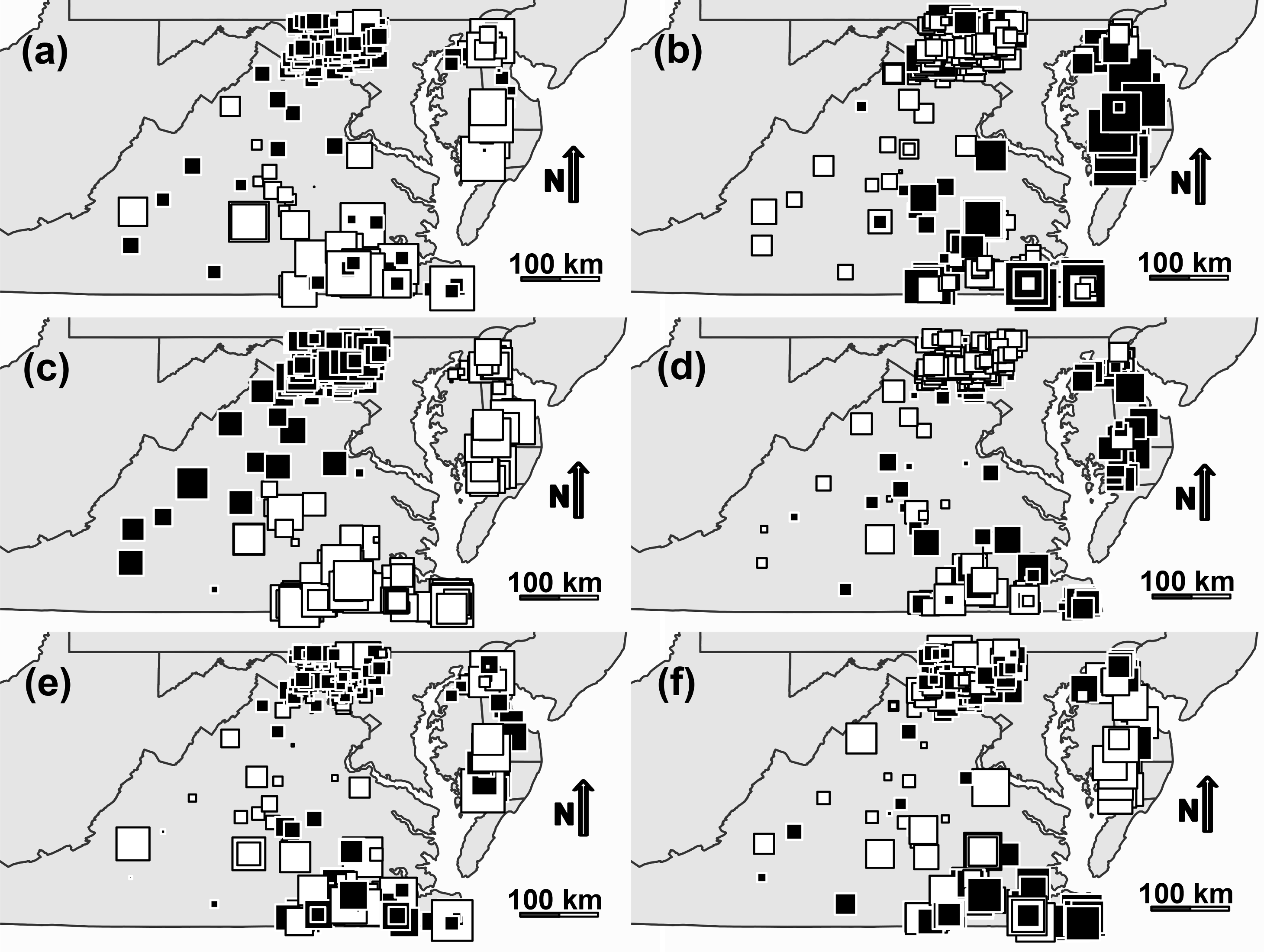

Supplement: S3 Fig — Maps of plot scores from the first two axes of PCA (a, b), approximation with explanatory variables through RDA (c, d), and the residual data analysis PRA (e, f). Analyses were performed on chi-square transformed soybean stink bug data emphasizing patterns in Chinavia hilaris and Euschistus servus from 329 soybean fields in mid-Atlantic USA. The black and the white squares indicate positive and negative plot scores respectively. The size of the squares is proportional to its score, the farther from zero being larger. (TIF) [file pone.0150649.s003.tif]
