# Supplementary material for: Contrasting Role of Temperature in Structuring Regional Patterns of Invasive and Native Pestilential Stink Bugs
Source: PLoS One. 2016 Feb 29;11(2):e0150649. doi: 10.1371/journal.pone.0150649 (PMC4771716; doi:10.1371/journal.pone.0150649)
Supplement: S1 Table — Description and summary (mean values, and standard deviation in parentheses) of the topographic and temperature variables at sampled soybean fields used for explaining stink bug distribution and abundance in mid-Atlantic US. (DOCX) [file pone.0150649.s004.docx]

Table S1. Description and summary (mean values, and standard deviation in parentheses) of the topographic and temperature variables at sampled soybean fields used for explaining stink bug distribution abundance in mid-Atlantic US.

| **Variable Code** | **Description** | **Mean (SD)** |
| --- | --- | --- |
| dist | Euclidean distance (km) of sampled fields from Allentown, Pennsylvania, proximal source of *Halyomorpha halys* populations | 270.0 (114.8) |
| elev | elevation (m) | 119.3 (86.4) |
| slope | percentage slope gradient | 3.0 (2.2) |
| sinasp | Sine of aspect value as a measure of 'Northness'. Values range from - 1 and 1 indicating South to North | 0.0 (0.7) |
| cosasp | Cosine of aspect value as a measure of 'Eastness'. Values range from - 1 and 1 indicating West to East | -0.1 (0.7) |
| mayT | average temperature for May (ºC) | 18.1 (1.5) |
| junT | average temperature for June (ºC) | 22.2 (1.4) |
| julT | average temperature for July (ºC) | 25.5 (1.0) |
| augT | average temperature for August (ºC) | 26.9 (1.0) |
