# Supplementary material for: Contrasting Role of Temperature in Structuring Regional Patterns of Invasive and Native Pestilential Stink Bugs
Source: PLoS One. 2016 Feb 29;11(2):e0150649. doi: 10.1371/journal.pone.0150649 (PMC4771716; doi:10.1371/journal.pone.0150649)
Supplement: S2 Table — Description and summary statistics of (mean values and standard deviation in parentheses) the land use variables at different spatial scales used for explaining stink bug distribution and abundance in mid-Atlantic US. (DOCX) [file pone.0150649.s005.docx]

Table S2. Description and summary statistics of (mean values and standard deviation in parentheses) the land use variables at different spatial scales used for explaining stink bug distribution and abundance in mid-Atlantic US.

| **Variable code** | **Description** | **Scale** | | | | | | |
| --- | --- | --- | --- | --- | --- | --- | --- | --- |
|  |  | 100m | 250m | 500m | 1 Km | 2.5 Km | 5 Km | 10 Km |
| cropscornsoy | percentage corn and soybean fields | 46.4 (25.1) | 18.1 (10.8) | 39.2 (20.9) | 31.4 (17.5) | 23.7 (14.0) | 20.7 (12.1) | 18.1 (10.8) |
| cropsother | percentage all other cultivated land use types | 2.7 (10.0) | 16.0 (8.4) | 2.9 (6.0) | 2.3 (4.4) | 2.3 (3.6) | 2.3 (2.5) | 16.0 (8.4) |
| forestdeci | percentage Deciduous forests - areas dominated by trees generally greater than 5 meters tall, and greater than 20perc. of total vegetation cover. More than 75perc. of the tree species shed foliage simultaneously in response to seasonal change. | 5.6 (9.9) | 31.6 (18.0) | 14.2 (12.5) | 19.3 (14.5) | 25.5 (17.3) | 27.6 (16.9) | 31.6 (18.0) |
| forestever | percentage Evergreen forests - areas dominated by trees generally greater than 5 meters tall, and greater than 20perc. of total vegetation cover. More than 75perc. of the tree species maintain their leaves all year. Canopy is never without green foliage. | 1.9 (7.1) | 6.8 (12.7) | 4.4 (11.0) | 5.4 (11.6) | 6.7 (13.4) | 6.8 (12.7) | 6.8 (12.7) |
| forestother | percentage all other natural habitat types | 7.7 (13.3) | 10.0 (15.6) | 5.6 (10.7) | 7.8 (13.8) | 9.6 (16.9) | 9.5 (15.1) | 10.0 (15.6) |
| develophigh | percentage Highly developed areas. Includes residential / work areas with impervious surfaces < 80 perc. of total area. Examples include apartment complexes, row houses and commercial/industrial. | 0.1 (0.9) | 26.2 (41.9) | 0.1 (0.4) | 0.1 (0.7) | 0.2 (0.8) | 0.2 (0.6) | 0.3 (0.4) |
| developmedium | percentage medium developed areas. Mix of constructed materials and vegetation. Impervious surfaces 50 - 79 perc. of total cover. Commonly single-family housing units | 0.5 (2.7) | 86.4 (88.5) | 0.5 (1.6) | 0.5 (1.4) | 0.6 (1.5) | 0.8 (1.5) | 0.9 (1.1) |
| developlow | percentage low development areas. Mix of constructed materials and vegetation. Impervious surfaces 20 - 49 perc. of total cover. Commonly single-family housing units | 5.4 (9.8) | 3.0 (3.0) | 2.5 (4.8) | 2.4 (4.4) | 2.5 (4.0) | 2.8 (3.8) | 3.0 (3.0) |
| developopen | percentage developed open areas. Some constructed materials, but mostly vegetation in the form of lawn grasses. Impervious surfaces < 20 perc. of total cover. Commonly include large-lot single-family housing units, vegetation planted in developed settings | 18.3 (14.7) | 7.2 (3.5) | 8.3 (6.4) | 6.9 (4.7) | 6.8 (4.7) | 7.3 (4.3) | 7.2 (3.5) |

Land use and crop acreage data were obtained from CropScape [46, 47]. More details on the crop data layers from CropScape are found in the metadata for each State ([www.nass.usda.gov/research/Cropland/metadata/meta.htm](http://www.nass.usda.gov/research/Cropland/metadata/meta.htm)). The descriptions for other land use data layers (forest cover and developed areas) were obtained from National Land Cover Dataset (NLCD) metadata (<http://www.mrlc.gov/nlcd11_leg.php>).
